# Chronic Cholesterol Exposure Disrupts Macrophage Polarization and Cytokine Secretion in a 3D Microenvironment

**DOI:** 10.1021/acsomega.5c09175

**Published:** 2026-02-05

**Authors:** Aliyaa Ali Alzaabi, Dheyab Saleh Abubaker, Jiranuwat Sapudom, Yamanappa Hunashal, Fabio Piano, Jeremy Teo

**Affiliations:** † Laboratory for Immuno Bioengineering Research and Applications, Division of Engineering, 167632 New York University Abu Dhabi, Abu Dhabi 129188, United Arab Emirates; ‡ Department of Biomedical Engineering, Tandon School of Engineering, New York University, New York, New York 11201, United States; § Biology Program, Science Division, 5894 New York University Abu Dhabi, Abu Dhabi 129188, United Arab Emirates; ∥ Center for Genomics and Systems Biology, New York University Abu Dhabi, Abu Dhabi 129188, United Arab Emirates; ⊥ Department of Biology, Center for Genomics and System Biology, New York University, New York, New York 11201, United States; # Department of Mechanical Engineering, Tandon School of Engineering, New York University, New York, New York 11201, United States

## Abstract

Cholesterol is essential for membrane organization and signaling, but excess cholesterol is increasingly linked to immune dysregulation. How chronic cholesterol loading shapes macrophage differentiation and polarization remains unclear. Here, we examined the effects of sustained cholesterol exposure on THP-1 monocytes and their polarization within 3D collagen hydrogels. At the monocyte stage, cholesterol caused cytotoxicity above 3 mg/mL, with an early decline in reactive oxygen species and metabolic remodeling marked by cholesterol accumulation, tricarboxylic acid cycle suppression, and redox imbalance. Subcytotoxic doses preserved cell count but altered metabolic profiles, indicating a primed state. Differentiation into uncommitted M0 macrophages produced only minimal phenotypic changes, though modest increases in IL-10, IFN-γ, and IP-10 suggested early functional effects. Under M1 polarization, cholesterol-loading macrophages showed reduced expression of CD80, CD86, and HLA-DR, yet secreted higher levels of both pro-inflammatory (IL-12p70, IFN-γ, IL-17A, MCP-1, IL-2) and regulatory (IL-10, IL-4) cytokines. Under M2 polarization, canonical markers CD206, CD105, and CD163 were diminished, while secretion of TGF-β1, IL-10, TNF-α, and IL-12p70 was increased. Across both conditions, cholesterol consistently uncoupled surface phenotype from cytokine output, producing a noncanonical hypersecretory state. These findings suggest that cholesterol primes monocytes and disrupts macrophage polarization, redirecting them toward mixed, hypersecretory phenotypes independent of stimulus. This work links cholesterol-induced metabolic stress to altered macrophage plasticity, with implications for maladaptive immune responses in cholesterol-rich environments.

## Graphical abstract

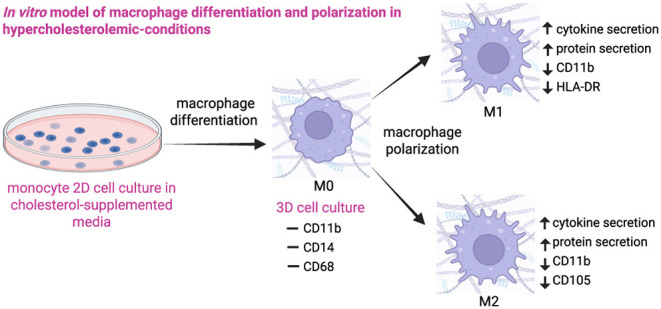

## 1 Introduction

Cholesterol is a structurally indispensable lipid that plays multifaceted roles in cellular physiology. It not only maintains membrane fluidity and supports lipid raft formation essential for receptor signaling but also serves as a biosynthetic precursor for steroid hormones, bile acids, and vitamin D.^1,2^ Under homeostatic conditions, cholesterol levels are tightly regulated by a dynamic balance between endogenous synthesis, dietary absorption, systemic transport via lipoproteins, and cellular uptake and efflux.^1,3^ However, metabolic dysregulation driven by genetic predisposition, dietary excess, and sedentary behavior disrupts this equilibrium, leading to hypercholesterolemia.^4,5^ This condition is a well-established risk factor for atherosclerosis and is closely associated with obesity, metabolic syndrome, and type 2 diabetes. Beyond its cardiovascular implications, accumulating evidence highlights cholesterol as a critical modulator of immune function.^6,7^ Clinically, hypercholesterolemia is linked to heightened susceptibility to infections, delayed wound healing, exacerbated inflammatory responses, and poor outcomes in conditions such as sepsis and COVID-19.^8^ These observations suggest that cholesterol overload perturbs immune cell function, although the underlying mechanisms remain incompletely defined.

Among immune cells, macrophages occupy a central role in orchestrating inflammation, tissue repair, and pathogen clearance. Derived from circulating monocytes, macrophages display remarkable phenotypic plasticity, classically spanning pro-inflammatory M1 to tissue-reparative M2 states. M1 macrophages, typically induced by IFN-γ and LPS, secrete cytokines such as TNF-α, IL-1β, and IL-12, whereas M2 macrophages, polarized by IL-4 or IL-13, exert anti-inflammatory and pro-repair functions through IL-10 and TGF-β1.^9,10^ In hyperlipidemic conditions, this polarization axis becomes dysregulated.^11,12^ Macrophages exposed to free or oxidized cholesterol exhibit altered receptor expression, reduced phagocytic capacity, and aberrant cytokine secretion.^13^ Moreover, intracellular cholesterol accumulation activates the NLRP3 inflammasome and drives a metabolic shift toward aerobic glycolysis, further amplifying unresolved inflammation.^14^

Beyond biochemical stimuli, macrophage phenotype and function are profoundly shaped by the physical properties of the extracellular matrix (ECM), including stiffness, dimensionality, and composition.^9^ Conventional two-dimensional (2D) culture systems, although widely used, fail to recapitulate the complexity of in vivo architecture and mechanical confinement. In 2D, cells exhibit distorted cytoskeletal organization, abnormal polarization, and misregulated signaling cascades, which may confound interpretation of lipid-mediated immune responses.^15,16^ This is particularly relevant when studying cholesterol–immune interactions, as lipid metabolism and matrix mechanics likely converge to influence macrophage fate decisions. Three-dimensional (3D) hydrogel-based culture systems more faithfully mimic native tissue environments by providing spatial organization and physiologically relevant mechanical cues.^17,18^ Such models offer a robust platform for dissecting immune–metabolic crosstalk under controlled conditions.

Building on this rationale, we investigated how sustained cholesterol exposure alters monocyte-to-macrophage differentiation and subsequent polarization in a 3D microenvironment. Using a collagen-based hydrogel model that recapitulates ECM architecture, THP-1 monocytes were subjected to prolonged cholesterol exposure, followed by quantification of intracellular lipid accumulation, metabolic activity, and reactive oxygen species (ROS) production. Subsequently, cells were polarized toward M1 or M2 phenotypes in the presence of cholesterol to simulate lipid-rich tissue environments. Through multiparametric phenotypic profiling, cytokine secretion analysis, and unsupervised clustering, this study seeks to elucidate how excess cholesterol reprograms macrophage heterogeneity and disrupts immune plasticity in metabolically stressed microenvironments.

## 2 Materials and Methods

### 2.1 Monocyte Culture and Cholesterol Treatment

The human monocytic cell line THP-1 (ATCC, Manassas, VA, USA) was maintained in RPMI-1640 medium supplemented with 10% fetal bovine serum (FBS), 1% HEPES, 1% sodium pyruvate, 0.1% β-mercaptoethanol, and 1% penicillin/streptomycin (all from Gibco, Thermo Fisher Scientific, Waltham, MA, USA). Cells were cultured at 37 °C in a humidified incubator with 5% CO_4_ under standard conditions.

For cholesterol conditioning, THP-1 cells were cultured with cholesterol (Oakwood Chemical, Estill, SC, USA), which was directly dissolved in complete RPMI-1640 medium and sterile-filtered prior to use, at concentrations ranging from 0 mg/mL (control) to 10 mg/mL for three consecutive passages. Passaging was performed every 3 days, prior to analysis or induction of macrophage differentiation. A conditioning period of three passages was selected to ensure sufficient cellular adaptation to the cholesterol-enriched environment, thereby minimizing acute stress responses and enabling the assessment of stable, cholesterol-driven immunometabolic reprogramming, mimicking long-term exposure to cholesterol.

### 2.2 Cell Count and Reactive Oxygen Species (ROS) Detection Using DHR123

Intracellular reactive oxygen species (ROS) levels were quantified using dihydrorhodamine 123 (DHR123; Thermo Fisher Scientific, Waltham, MA, USA), a cell-permeable, nonfluorescent dye that is oxidized to fluorescent rhodamine 123 in the presence of ROS. THP-1 cells previously conditioned with cholesterol (0–10 mg/mL) for three consecutive passages were harvested and resuspended at 1 × 10^6^ cells/mL in RPMI-1640 medium. Cells were incubated with 5 μM DHR123 at 37 °C for 30 min in the dark, washed with PBS, and resuspended in PBS.

Fluorescence intensity of oxidized rhodamine 123 was measured using the Attune NxT Flow Cytometer (Thermo Fisher Scientific, Waltham, MA, USA) with excitation at 488 nm and emission collected at 530 nm (FITC channel). In parallel, cell count was assessed directly from flow cytometry. This ensured that ROS measurements reflected viable cell populations and allowed normalization of fluorescence to cell counts. Data were analyzed using FlowJo software (BD Biosciences, San Jose, CA, USA), and ROS levels were quantified based on geometric mean fluorescence intensity (gMFI). Cell counts were reported as absolute event numbers per sample, normalized to untreated controls. Experiments were performed in four replicates.

### 2.3 NMR-Based Metabolomic and Lipidomic Profiling

Intracellular metabolites and lipids were extracted using a modified Folch extraction protocol based on the methanol–chloroform–water method.^19^ Cell pellets containing 1 × 10^7^ THP-1 cells were resuspended in 500 μL of ice-cold methanol (Sigma-Aldrich, St. Louis, MO, USA), followed by the addition of 500 μL of chloroform (Sigma-Aldrich, St. Louis, MO, USA). After thorough vortexing, 200 μL of ultrapure water (Millipore, Burlington, MA, USA) was added to induce phase separation. Samples were centrifuged at 3000 × *g* for 5 min at 4 °C. The resulting biphasic mixture was separated into an upper aqueous phase (containing polar metabolites) and a lower organic phase (containing lipids), each of which was carefully collected. Extracted fractions were dried under vacuum using an Evaporator HT-12 3i system (Genevac Ltd., Ipswich, UK) or evaporated under a gentle nitrogen stream prior to downstream analysis.

For NMR analysis, dried aqueous extracts were reconstituted in 500 μL of 100 mM phosphate buffer in D_4_O (pH 7.4) containing 0.2 mM 3-(Trimethylsilyl)-1-propanesulfonic acid-*d*_6_ sodium salt (DSS-*d*_6_) as an internal standard (Sigma-Aldrich, St. Louis, MO, USA), while lipid extracts were dissolved in 500 μL of deuterated chloroform (Sigma-Aldrich, St. Louis, MO, USA). One-dimensional ^1^H NMR spectra were acquired using Bruker Avance III HD 600 MHz (Bruker) using the “noesypr1d” pulse sequence. For aqueous samples, presaturation was applied during the relaxation delay and mixing time, whereas spectra of lipid samples in chloroform were recorded without presaturation. The acquisition parameters were: spectral width of 12 ppm, relaxation delay of 5 s, acquisition time of 4 s, and a mixing time of 100 ms, while two-dimensional ^1^H–^1^H TOCSY was conducted with the DIPSI2 sequence along with water suppression achieved by excitation sculpting with gradients by setting 2k × 128 time domain data points, 128 transients per FID, a relaxation delay of 2.0 s, and a TOCSY mixing time of 100 ms to confirm the metabolite assignments.^20,21^ All the NMR data were processed using TOPSPIN 4.4.1 software (Bruker), and metabolites were identified by comparing spectra with reference databases from Chenomx NMR Suite V11.0 Professional (Chenomx Inc., Edmonton, Canada), BBIOREFCODE-2.7.0 (Bruker Biospin, Rheinstetten, Germany), and the Human Metabolome Database (HMDB).^22^ Metabolite quantification was performed using internal standard DSS-*d*_6_, and changes in metabolite levels were calculated to capture condition-specific metabolic variations. Quantitative and functional analysis of the obtained data was performed using MetaboAnalyst 6.0.^23^ Experiments were performed in three replicates.

### 2.4 Fabrication of Three-Dimensional Collagen Hydrogels

Three-dimensional (3D) collagen matrices with a final collagen concentration of 2 mg/mL were prepared as previously described.^24^ Briefly, type I rat tail collagen (Advanced BioMatrix, Carlsbad, CA, USA) was mixed with 250 mM phosphate buffer and 0.1% acetic acid (both from Sigma-Aldrich, St. Louis, MO, USA) to achieve the desired concentration. A total of 50 μL of the collagen solution was dispensed onto glutaraldehyde-coated coverslips (VWR International, Radnor, PA, USA) and allowed to polymerize in a humidified incubator at 37 °C. The collagen hydrogels were subsequently stored in phosphate-buffered saline (PBS; Gibco, Thermo Fisher Scientific, Waltham, MA, USA) until use in cell culture experiments. Our collagen matrix yields a stiffness of approximately 150 Pa, and an average pore size of approximately 8 μm.^25^

### 2.5 Macrophage Differentiation and Polarization

The human monocytic cell line THP-1 (ATCC, Manassas, VA, USA) was cultured in RPMI-1640 medium supplemented with 10% fetal bovine serum (FBS), 1% sodium pyruvate, 0.01% β-mercaptoethanol, and 1% penicillin–streptomycin (all from Gibco, Thermo Fisher Scientific, Waltham, MA, USA) at 37 °C in a humidified atmosphere containing 5% CO_4_. The differentiation and activation protocols for THP-1-derived macrophages were established according to a previously published method.^9^ Briefly, THP-1 cells were seeded onto reconstituted three-dimensional (3D) collagen matrices and differentiated into uncommitted macrophages (M0) using 300 nM phorbol 12-myristate 13-acetate (PMA; Sigma-Aldrich, St. Louis, MO, USA) in RPMI-1640 medium without FBS. After 6 h of stimulation, the PMA-containing medium was removed, cells were washed with phosphate-buffered saline (PBS; Gibco, Thermo Fisher Scientific, Waltham, MA, USA), and rested for 24 h in fresh RPMI-1640 medium without FBS or PMA. Activation media and resting media were supplemented with cholesterol for cells grown in cholesterol-conditioned conditions.

For polarization, cells were subsequently activated for 48 h under pro-inflammatory (M1) conditions using 500 pg/mL lipopolysaccharide (LPS; Sigma-Aldrich, St. Louis, MO, USA) and 20 ng/mL interferon-gamma (IFN-γ; BioLegend, San Diego, CA, USA), or under anti-inflammatory (M2) conditions using 20 ng/mL interleukin-4 (IL-4; BioLegend, San Diego, CA, USA) and 20 ng/mL interleukin-13 (IL-13; BioLegend, San Diego, CA, USA). Activation media was supplemented with cholesterol for cells grown in cholesterol-conditioned conditions, maintaining cholesterol exposure throughout differentiation and polarization.

### 2.6 Immunophenotyping of THP-1-Derived Macrophages

To characterize cell surface marker expression, macrophages were recovered from the 3D collagen matrices by enzymatic digestion using collagenase (2 mg/mL, Advanced Biomatrix, Carlsbad, CA, USA) prepared in culture medium and incubated for 10 min under standard culture conditions (37 °C, 5% CO_4_). Cells were then stained for 30 min on ice with fluorochrome-conjugated monoclonal antibodies, as listed in Supplementary Table 1. For intracellular staining of NFκB p50, STAT3 (Tyr705), and STAT6 (Tyr641) the cells were then fixed with 4% PFA, permeabilized with 0.1% Triton X-100, and blocked with 1% bovine serum albumin (BSA) prior to staining with antibodies. Antibodies were diluted 1:500 in complete cell culture medium. To exclude nonviable cells from analysis, DRAQ7 (BioLegend, San Diego, CA, USA) was added at a 1:2000 dilution. All antibodies and viability dyes were obtained from BioLegend (San Diego, CA, USA). Samples were analyzed using an Attune NxT Flow Cytometer equipped with an autosampler (Thermo Fisher Scientific, Waltham, MA, USA).

Flow cytometry data were analyzed using FlowJo software v10.8.1 (BD Biosciences, San Jose, CA, USA). Dimensionality reduction was performed using the Uniform Manifold Approximation and Projection (UMAP) plugin, which provides two-dimensional visualization of high-dimensional cytometry data. Clustering was performed using the FlowSOM plug-in, which applies a self-organizing map algorithm to group cells based on marker expression patterns. The combination of UMAP visualization and FlowSOM clustering enabled the unsupervised identification of five macrophage clusters, each defined by distinct surface marker signatures. Experiments were performed in six replicates.

### 2.7 Cytokine Quantification Using Multiplex Bead-Based Immunoassay

To assess cytokine secretion, cell culture supernatants were collected following macrophage activation. Cytokine concentrations (IFN-γ, IL-1β, IL-2, IL-4, IL-6, IL-8, IL-10, IL-12p70, IL-17A, IP-10, MCP-1, TGF-β1, and TNF-α) were quantified using a bead-based multiplex immunoassay (LEGENDplex, BioLegend, San Diego, CA, USA), according to the manufacturer’s instructions. Samples were analyzed using an Attune NxT Flow Cytometer equipped with an autosampler (Thermo Fisher Scientific, Waltham, MA, USA), and data were processed using LEGENDplex analysis software (BioLegend, San Diego, CA, USA). Experiments were performed in six replicates.

In addition to cytokine quantification, total protein quantification of M0, M1, and M2 macrophage supernatant was quantified using NanoDrop direct absorbance measurements at 280 nm. Experiments were performed in at least 4 replicates.

### 2.8 Statistical Analysis

All experiments were performed in at least three biological replicates unless otherwise stated. Data are presented as mean ± standard deviation (SD). For comparisons between two groups, an unpaired Student’s *t*-test was used. For comparisons involving more than two groups, one-way ANOVA followed by Tukey’s post hoc test was applied. Analyses were conducted using GraphPad Prism 10 (GraphPad Software, San Diego, CA, USA). Significance levels are indicated as follows: *p* < 0.05 (*), *p* < 0.01 (**), *p* < 0.001 (***), and *p* < 0.0001 (****).

## 3 Results and Discussion

This study aims to demonstrate how chronic cholesterol exposure alters the monocyte-to-macrophage axis within a 3D microenvironment, uncoupling canonical polarization programs from functional cytokine output. To better mimic the tissue conditions encountered by macrophages in vivo, we employed a 3D collagen matrix that provides physiologically relevant spatial and mechanical cues. Unlike previous studies that acutely expose differentiated macrophages to modified lipids, our model subjected THP-1 monocytes to chronic exposure of soluble, unesterified (free) cholesterol throughout their differentiation and polarization.^26^ This approach reflects the sustained lipid stress. The use of free cholesterol, rather than modified lipoproteins such as acetylated LDL (AcLDL)^25^ or oxidized LDL (oxLDL),^27^ was a deliberate choice. While AcLDL and oxLDL offer greater physiological relevance in modeling foam cell formation, their effects are largely mediated through receptor-specific uptake. In contrast, our model allowed us to decouple cholesterol uptake from receptor-mediated signaling and isolate the effects of unregulated intracellular cholesterol levels. This design provides a clearer view of how persistent cholesterol stress alone can reshape macrophage phenotype and immune function.

### 3.1 Cholesterol Overload Induces Dose-Dependent Redox Impairment Preceding Cell Count Decline in THP-1 Monocytes

To establish a physiologically relevant model for chronic cholesterol exposure, we developed an in vitro system that recapitulates key aspects of monocyte-to-macrophage differentiation and polarization. As illustrated in Figure 1A, circulating monocytes encounter cholesterol-rich environments in vivo during circulation in the blood and extravasation into tissues, where they differentiate into macrophages and subsequently polarize in response to microenvironmental cues. To capture this process experimentally, THP-1 monocytes were exposed to unesterified cholesterol in two-dimensional (2D) culture for three passages then, embedded in a three-dimensional (3D) collagen hydrogel and differentiation using PMA, then polarization toward M1 (LPS/IFNγ) or M2 (IL-4/IL-13) phenotypes in the presence or absence of cholesterol. This workflow enabled us to investigate how excess cholesterol influences macrophage fate within a controlled, tissue-mimetic microenvironment. Bright-field microscopy showed that THP-1 monocytes maintained a comparable overall morphology regardless of cholesterol treatment (Figure 1B). Both control and cholesterol-exposed cells remained evenly distributed, and no overt morphological alterations were detected at this stage. This indicates that cholesterol exposure does not immediately impact cell appearance, and functional changes must instead be evaluated at the metabolic and viability level.

**Figure 1:**
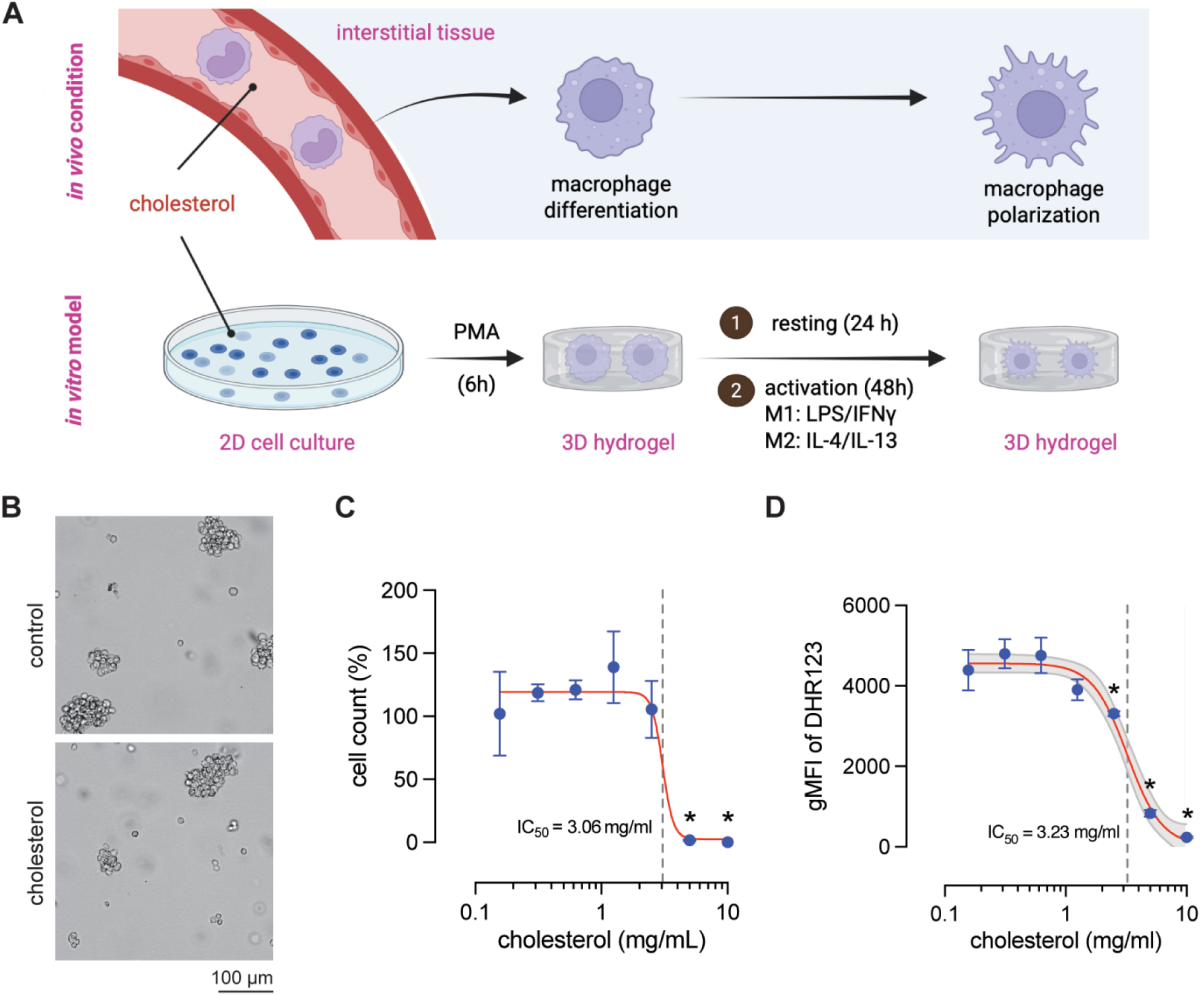
Experimental design and long-term cholesterol conditioning in THP-1 monocytes. (A) Schematic overview of the in vivo relevance of cholesterol exposure and the in vitro experimental workflow. Cholesterol accumulation in interstitial tissues influences monocyte-to-macrophage differentiation, followed by polarization into M1 (LPS/IFNγ) or M2 (IL-4/IL-13) phenotypes. Cholesterol is present in all activation, resting, and polarization media. (Scale bar = 100 μm) (B) Representative bright-field images of THP-1 monocytes after cholesterol exposure over three passages. (C) Cell count of THP-1 monocytes across a range of cholesterol concentrations. (D) Geometric mean fluorescence intensity (gMFI) of DHR123 as a measure of intracellular ROS following cholesterol exposure. Shaded area represents 95% confidence interval. For Figure 1C,1D, 0 mg/mL condition was represented as 0.01 mg/mL to allow plotting on the logarithmic *x*-axis. Data are presented as mean ± SD. Experiments were perfromed at least in 4 independent replicates.

To quantify cholesterol-induced toxicity, cell counts were measured across a concentration range of 0.1–10 mg/mL. As shown in Figure 1C, cell count was preserved between 0.1 and 2 mg/mL but declined sharply in a nonlinear fashion above 3 mg/mL. The cytotoxic threshold was defined at 3.06 mg/mL, at which THP-1 monocytes could no longer sustain homeostasis. This value lies close to the range of total plasma cholesterol concentrations reported in hypercholesterolemia, approximately 2.59 mg/mL in children and 2.90 mg/mL in adults.^28^ According to a study involving 12.8 million adults, the total cholesterol range associated with the lowest mortality was between 2.1 and 2.49 mg/mL.^29^ These comparisons provide a useful benchmark but should be considered conceptual rather than direct, since circulating cholesterol is predominantly lipoprotein-bound in vivo. In our cell culture system, although a portion of cholesterol likely associates with serum proteins in FBS, the binding capacity is limited compared to human plasma, leaving a larger freely bioavailable pool accessible to immune cells.^30^ Given that freely bioavailable cholesterol can perturb mitochondrial integrity and redox balance, we next analyzed oxidative metabolism using DHR123 staining as a surrogate for intracellular ROS. Unexpectedly, ROS levels began to decline at 2.5 mg/mL cholesterol, before the major loss of cell count at 3.06 mg/mL (Figure 1D). The decline in ROS was more gradual than the drop in cell count. This finding contrasts with previous reports describing cholesterol-driven ROS increases via mitochondrial stress and NADPH oxidase activation.^31,32^ The early reduction observed here most likely reflects metabolic adaptation. At higher concentrations (>2.5 mg/mL), ROS levels dropped sharply in parallel with cell count, consistent with the loss of metabolically competent cells (Figure 4D).

These results demonstrate that cholesterol exerts a biphasic effect on THP-1 monocytes: subcytotoxic concentrations (<3 mg/mL) preserve cell count but impair redox homeostasis, whereas higher levels (>2.5 mg/mL) induce metabolic collapse and cell death. We therefore selected 2.5 mg/mL for subsequent experiments, as this concentration lies just below the cytotoxic threshold, ensuring cell survival while imposing sufficient metabolic stress to model the chronic lipid burden encountered in pathophysiological settings.

### 3.2 Exogenous Cholesterol Uptake Is Associated with Remodeling of Cholesterol Pools and Metabolic Stress

To confirm that THP-1 monocytes internalize exogenous cholesterol at the selected concentration (2.5 mg/mL), we performed intracellular lipid profiling using 1D ^1^H nuclear magnetic resonance (^1^H NMR) spectroscopy. This method allows quantitative assessment of cellular lipids, including cholesterol and fatty acids.^33,34^ Representative spectra (Figure 2A) revealed signals corresponding to unsaturated fatty acids (UFAs), free cholesterol (FC), and total cholesterol (TC). Quantitative analysis (Figure 2B) showed that cholesterol-treated cells accumulated significantly higher levels of FC and TC and exhibited an increased TC/FC ratio, while UFA levels remained unchanged. FC represents the unesterified, biologically active form of cholesterol integrated into membranes, whereas TC includes both free and esterified cholesterol. The TC/FC ratio therefore reflects the balance between active and stored cholesterol pools; an increased ratio indicates enhanced esterification and storage in lipid droplets, a process that helps buffer against FC-induced cytotoxicity.^35,36^ Elevated FC, however, is also expected to decrease membrane fluidity by increasing lipid packing, which can impair membrane protein function, signaling, and mitochondrial integrity.^37^ These changes are consistent with the early alterations in redox homeostasis and cell count observed in Figure 1 and suggest that the FC–TC balance might play a role in determining cellular tolerance to cholesterol loading. The stability of UFA levels despite cholesterol accumulation is biologically significant. UFAs are essential membrane components and precursors for lipid mediators regulating inflammation and cell signaling.^38,39^ Their preservation suggests that cholesterol loading induces selective remodeling of cholesterol pools without broadly disrupting phospholipid composition.

**Figure 2:**
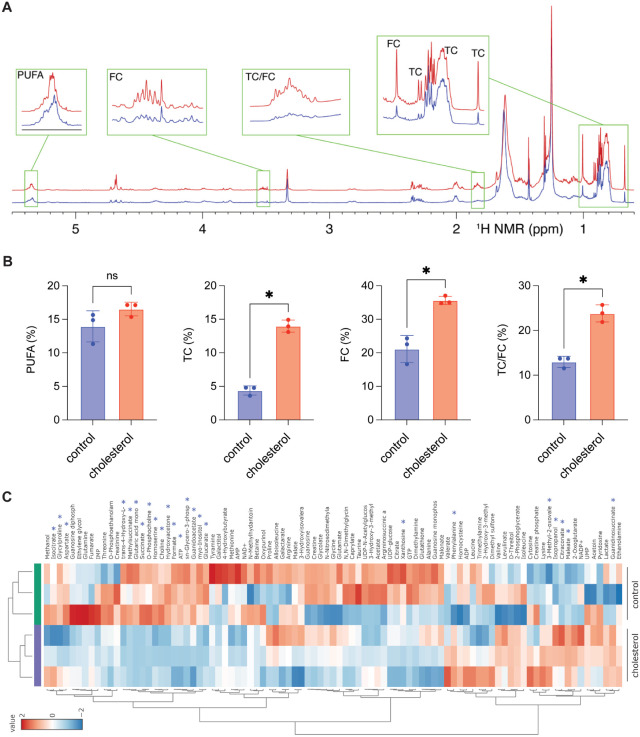
Lipid and metabolite profiles of monocytes after long-term cholesterol exposure assessed by ^1^H NMR. (A) Representative ^1^H NMR spectra of lipid extracts from control (blue) and cholesterol-treated (2.5 mg/mL; red) cells, showing regions corresponding to unsaturated fatty acids (UFA), free cholesterol (FC), and total cholesterol (TC). (B) Quantification of intracellular UFA, TC, FC, and the TC/FC ratio. Data are mean ± SD (C) Heatmap of intracellular metabolites in control and cholesterol-treated cells. Red indicates relative upregulation and blue indicates downregulation. Statistical analysis was performed using Student’s *t*-test; *p* < 0.05 (*), *p* < 0.01 (**), *p* < 0.001 (***), *p* < 0.0001 (****). Experiments were performed in triplicates.

To determine whether cholesterol-induced lipid alterations were accompanied by changes in central metabolism, we next applied ^1^H NMR based metabolomic profiling, an approach that provides insight into central metabolic pathways, including those associated with energy metabolism and mitochondrial function.^40,41^ As shown in Figure 2C, cholesterol-treated cells exhibited marked reductions in tricarboxylic acid (TCA) cycle intermediates (citrate, isocitrate, succinate, fumarate, malate), NAD^+^, glutathione, and amino acids linked to mitochondrial metabolism (glutamate, glutamine, aspartate).^42,43^ Supplementary Figure S1 is added for better visualization of Figure 2C. Energy metabolites were also perturbed, with depletion of ATP and AMP alongside accumulation of ADP and phosphocreatine, suggesting impaired oxidative phosphorylation and altered energy balance.^44^ Elevated lactate levels indicated compensatory glycolytic activation. Consistent with this interpretation, our metabolic and bioenergetic analyses indicate that cholesterol exposure induces metabolic reprogramming rather than overt mitochondrial damage. Specifically, we observed a reduction in TCA cycle intermediates, ROS, accompanied by decreased ATP levels (Supplementary Figure S2). While changes in proton leak, assessed indirectly through mitochondrial membrane potential using the JC-1 assay, were minor (Supplementary Figure S3), it suggests that no major damage occurred to the mitochondrial membranes following cholesterol exposure. Together, our findings correlate with previous findings to indicate that cholesterol accumulation does not cause widespread mitochondrial injury but instead triggers a metabolically adaptive response.^45^

### 3.3 Cholesterol Preloading Minimally Alters M0 Macrophage Differentiation

Having established that cholesterol preloading induces lipid accumulation and metabolic stress (Figure 2), we next investigated whether these changes affect the ability of monocytes to differentiate into macrophages and the phenotypic diversity of the resulting M0 populations. This question is biologically relevant because circulating monocytes in hypercholesterolemic conditions display altered transcriptional programs in cholesterol transport and inflammatory pathways, predisposing them toward atypical differentiation trajectories.^46^ To model this process under physiologically relevant conditions, THP-1 monocytes were exposed to cholesterol, subsequently infiltrated into 3D collagen matrices then differentiated into uncommitted M0 macrophages using PMA stimulation, in the presence of cholesterol. The 3D collagen matrices, provide a tissue-like microenvironment for macrophage differentiation. As shown in Figure 3A, both control and cholesterol-loaded macrophages embedded in 3D collagen hydrogels appeared predominantly round and nonspread, consistent with an unpolarized M0 morphology.

**Figure 3:**
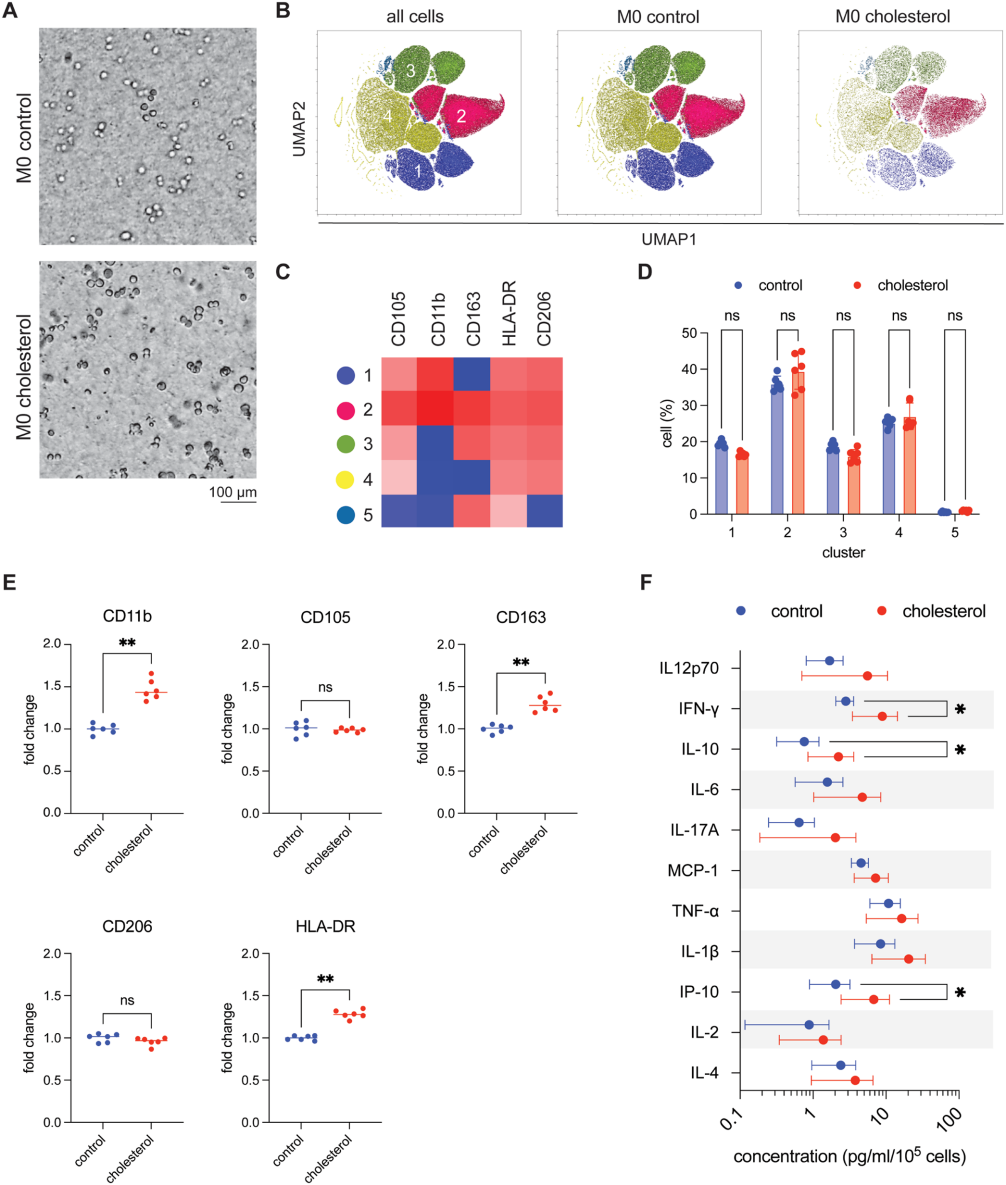
Effects of monocyte cholesterol preloading on subsequent M0 macrophage differentiation. (A) Representative phase-contrast micrographs of M0 macrophages embedded in 3D collagen hydrogels under control and cholesterol-treated conditions. (Scale bar = 100 μm). (B) Comparison of CD11b, CD14, and CD68 expression between control and cholesterol-treated THP-1 monocytes and M0 macrophages. Data are normalized to untreated THP-1 controls. (C) Single-cell UMAP projections of flow cytometry data with separate views for control and cholesterol-treated groups. (D) Heatmap showing relative expression of CD11b, CD105, CD163, CD206, and HLA-DR across five macrophage clusters. (E) Quantification of cluster proportions in control versus cholesterol-treated conditions. (F) Cytokine secretion profiles measured from M0 macrophage culture supernatants by bead-based multiplex immunoassay. Data are presented as mean ± SD. Significance levels are indicated as follows: *p* < 0.05 (*), *p* < 0.01 (**), *p* < 0.001 (***), and *p* < 0.0001 (****). Experiments were performed in 6 replicates.

To examine macrophage subpopulations within the 3D collagen matrix, we first validated THP-1 to M0 differentiation by assessing the macrophage markers CD11b, CD14, and CD68. Representative histogram plots of these markers are shown in Supplementary Figure S4. We next analyzed surface marker expression at the single-cell level. Unsupervised clustering of flow cytometry data identified five subsets, visualized by UMAP (Figure 3B–D). Cholesterol loading resulted in no changes in the proportions of clusters (Figure 3E). Both the cholesterol and control conditions are composed mainly of cluster 2. It is distinguished by uniformly high expression of CD11b, CD105, CD163, CD206, and HLA-DR. To validate these observations at the population level, we next quantified surface marker expression across the macrophage population (Supplementary Figure S5). Cholesterol-loaded macrophages displayed significant upregulation of CD163, and HLA-DR, whereas CD105 and CD206 remained unchanged. These bulk measurements complement the single-cell analysis: while cluster 2 displayed uniformly high expression of all five markers, its modest expansion meant that only a subset of markers (CD11b, CD163, HLA-DR) shifted detectably at the population level, whereas CD105 and CD206 remained buffered by stable expression across the other clusters. Finally, to determine whether these phenotypic shifts were accompanied by functional changes, we analyzed cytokine secretion profiles (Figure 3F). Cholesterol-loaded macrophages secreted higher levels of IL-10, IFN-γ, and IP-10, while other cytokines (IL-12p70, IL-6, IL-17A, MCP-1, TNF-α, IL-1β, IL-2, and IL-4) remained unchanged. The simultaneous increase of IL-10, IFN-γ, and IP-10 indicates that cholesterol loading promotes a hybrid activation state even under baseline M0 conditions. This primed state suggests that prior cholesterol exposure may alter how macrophages subsequently respond to classical polarization cues, providing the rationale for examining M1 and M2 polarization under cholesterol-rich conditions

### 3.4 Cholesterol-Loaded Macrophages Show Reduced Surface Activation but Enhanced Cytokine Secretion under M1 Polarization

M1 macrophages are central effectors of host defense, characterized by high expression of costimulatory molecules (CD80, CD86), antigen presentation via HLA-DR, and secretion of pro-inflammatory cytokines such as IL-12, TNF-α, and IFN-γ. These functions are essential for pathogen clearance, antitumor immunity, and shaping T cell responses.^6,47^ In metabolic diseases such as atherosclerosis, circulating monocytes encounter cholesterol-rich environments before and during their differentiation into macrophages.^48^ To determine the impact of cholesterol conditioning on inflammatory responsiveness, cholesterol-loaded M0 macrophages were stimulated with LPS and IFN-γ to induce M1 polarization in the 3D collagen environment.

Unsupervised clustering of single-cell flow cytometry data identified five macrophage subsets defined by TLR4, CD86, CD80, HLA-DR, CD11b, and CD83 (Figure 4B–C). In control of M1 macrophages, cluster 3 predominated and expressed high levels of all markers, consistent with a classical M1 phenotype. In contrast, cholesterol-loaded M1 macrophages showed a marked reduction in cluster 3 and redistribution into clusters 1 and 2 (Figure 4D). Cluster 1 expressed high CD80, CD83, and CD86 but reduced HLA-DR, CD11b, and TLR4, while cluster 2 showed moderate CD86/CD80 with otherwise low expression. These populations represent partially activated states, biased toward costimulatory molecules but lacking full antigen-presenting and innate-sensing features. Population-level analysis confirmed significantly reduced expression of CD11b, CD80, CD86, HLA-DR, TLR4, and CD83 in cholesterol-loaded M1 macrophages compared to controls (Figure 4E). This indicates impaired acquisition of canonical M1 surface features, particularly those linked to antigen presentation and pathogen recognition.

**Figure 4:**
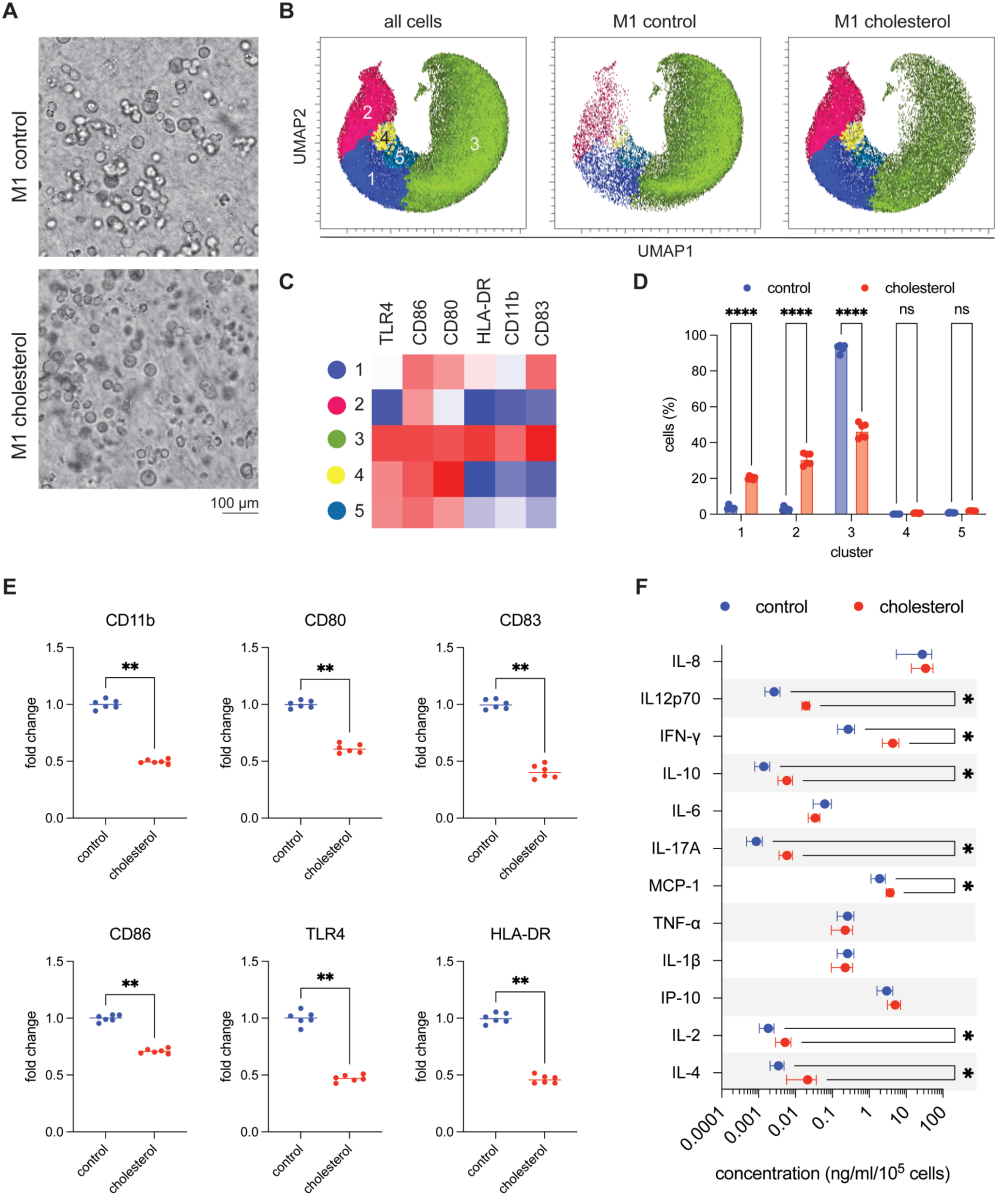
Immunophenotyping of M1-polarized macrophages derived from cholesterol-conditioned M0 THP-1 cells reveals distinct phenotypic remodeling. (A) Representative phase-contrast images of M1-polarized macrophages embedded in 3D collagen hydrogels, derived from control and cholesterol-treated M0 THP-1 cells. (Scale bar = 100 μm). (B) UMAP projections of single-cell flow cytometry data depicting clustering across all samples, with separate views for control and cholesterol-treated groups. (C) Heatmap illustrating the relative expression of surface markers (CD11b, CD80, CD83, CD86, TLR4, HLA-DR) across five identified macrophage clusters. (D) Quantification of the proportion of cells within each cluster in control versus cholesterol-treated conditions. (E) Surface marker expression profiles comparing control and cholesterol-treated M0 macrophages. Values were normalized to control. (F) Cytokine secretion profiles in M1 macrophage culture supernatants, as measured by bead-based multiplex immunoassay. Statistical significance was evaluated using Student’s *t*-test. Significance levels are indicated as follows: *p* < 0.05 (*), *p* < 0.01 (**), *p* < 0.001 (***), and *p* < 0.0001 (****). Experiments were performed in 6 replicates.

Cytokine profiling, however, revealed an opposite effect. Cholesterol-loaded M1 macrophages secreted higher levels of pro-inflammatory cytokines (IL-12p70, IFN-γ, IL-17A, MCP-1) and regulatory cytokines (IL-10, IL-4) compared to controls (Figure 4F). The increase in cytokine secretion despite reduced surface activation suggests a functional decoupling between phenotype and secretory activity. Given that clusters 1 and 2 expanded under cholesterol treatment, it is likely that these subsets contribute disproportionately to cytokine hypersecretion. Although less activated at the surface level, these clusters may be primed for enhanced intracellular signaling. Cholesterol is known to activate the NLRP3 inflammasome,^14^ reorganize lipid rafts to amplify TLR signaling,^49^ and trigger ER stress and ROS-driven MAPK/NF-κB pathways,^50^ all of which can enhance cytokine release independently of classical surface marker expression. Future studies examining caspase-1 activity, gasdermin D cleavage, and NLRP3 inhibition will help distinguish between inflammasome-dependent and stress-driven inflammasome-independent cytokine secretion.

In summary, cholesterol preloading shifts M1 polarization away from classical surface activation (cluster 3) and toward partially activated subsets (clusters 1 and 2) that hyper-secrete cytokines. This results in a noncanonical hybrid state, characterized by reduced expression of antigen-presenting and costimulatory markers yet increased secretion of both pro-inflammatory and regulatory mediators. Such decoupling of phenotype and function may have important consequences in vivo, as macrophages with impaired antigen presentation^51^ but heightened cytokine release through inflammasome and stress-related pathways^14,50^ could sustain chronic inflammatory signaling while providing limited support for adaptive immune responses. Similar phenotype–function mismatches have been reported in lipid-loaded macrophages where altered membrane organization suppresses receptor expression but enhances secretory activity.^49^

### 3.5 Cholesterol Loading Disrupts M2 Polarization and Drives a Hypersecretory Phenotype

M2 macrophages, typically induced by IL-4/IL-13, are associated with tissue repair and immunoregulation and are characterized by high expression of CD206 and CD105.^47^ To investigate whether cholesterol conditioning alters this program in a 3D environment, THP-1 monocytes were preloaded with cholesterol and subsequently polarized with IL-4 and IL-13.

As shown in Figure 5A, both control and cholesterol-loaded M2 macrophages remained viable in 3D collagen matrices with similar morphology to controls, indicating that cholesterol exposure did not prevent macrophage differentiation. Flow cytometry, however, revealed marked phenotypic shifts. Unsupervised clustering identified five subsets (Figure 5B,C). In control conditions, cluster 4 predominated, expressing high CD206 and CD105 together with CD163, consistent with a canonical M2 identity. Cholesterol loading led to a significant reduction of cluster 4 and expansion of clusters 3, and 5 (Figure 5D). Cluster 3 expressed intermediate CD206 and CD163 but reduced CD105, resembling an incomplete M2 state; cluster 5 displayed higher CD105 with reduced CD163, suggestive of a nonclassical regulatory phenotype. Collectively, these redistributions indicate that cholesterol disrupts canonical M2 polarization and promotes the emergence of incomplete or altered regulatory profiles. At the population level, cholesterol-loaded M2 macrophages exhibited reduced expression of CD11b, CD105, CD163, and CD206, while HLA-DR remained unchanged (Figure 5E). The loss of CD206 and CD105 suggests impaired tissue-repair capacity, whereas reduced CD163 indicates diminished immunoregulatory function. These impairments are consistent with previous reports that lipid accumulation interferes with macrophage polarization and scavenging activity.^50^

**Figure 5:**
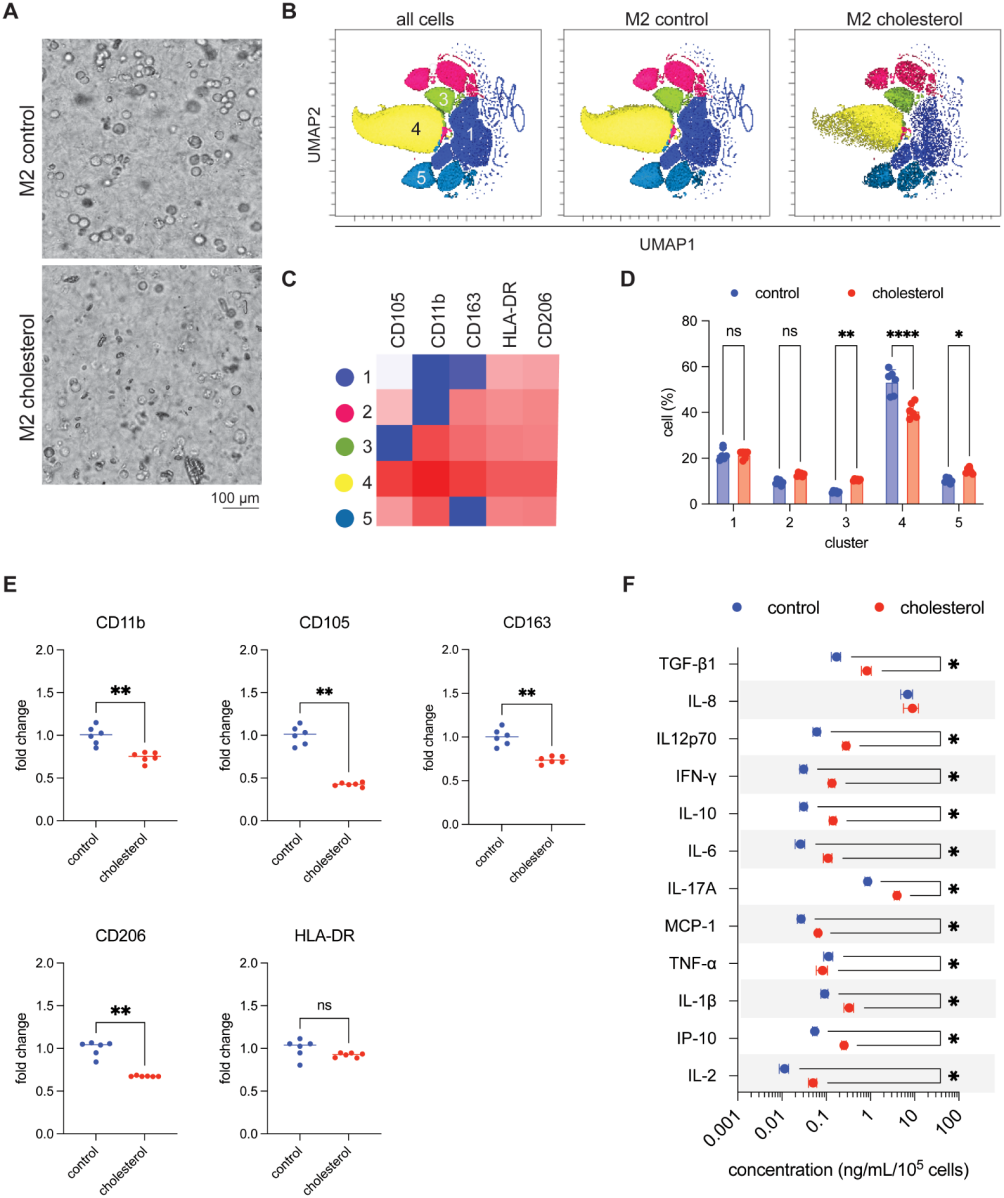
Immunophenotyping of M2-polarized macrophages derived from cholesterol-conditioned M0 THP-1 cells reveals distinct phenotypic remodeling. (A) Representative phase-contrast images of M2-polarized macrophages embedded in 3D collagen hydrogels, derived from control and cholesterol-treated M0 THP-1 cells. (Scale bar = 100 μm). (B) UMAP projections of single-cell flow cytometry data depicting clustering across all samples, with separate views for control and cholesterol-treated groups. (C) Heatmap illustrating the relative expression of surface markers (CD11b, CD105, CD163, CD206, HLA-DR) across five identified macrophage clusters. (D) Quantification of the proportion of cells within each cluster in control versus cholesterol-treated conditions. (E) Surface marker expression profiles comparing control and cholesterol-treated M0 macrophages. Values were normalized to control. (F) Cytokine secretion profiles in M2 macrophage culture supernatants, as measured by bead-based multiplex immunoassay. Statistical significance was evaluated using Student’s *t*-test. Significance levels are indicated as follows: *p* < 0.05 (*), *p* < 0.01 (**), *p* < 0.001 (***), and *p* < 0.0001 (****). Experiments were performed in 6 replicates.

Cytokine analysis revealed a striking divergence between surface phenotype and functional output. Cholesterol-loaded M2 macrophages secreted higher levels of TGF-β1 and IL-10, consistent with regulatory activity, but also significantly increased pro-inflammatory cytokines including IL-12p70, IFN-γ, IL-6, IL-17A, MCP-1, IL-1β, and IP-10 (Figure 5F). This paradoxical profile indicates that cholesterol does not reinforce a canonical M2 program but instead drives macrophages into a mixed activation state characterized by diminished surface marker identity and enhanced cytokine secretion, a hypersecretory phenotype. Physiologically, this may compromise wound repair (via reduced CD206/CD105) and limit resolution of inflammation (via reduced CD163). Pathologically, the simultaneous elevation of regulatory and inflammatory cytokines (e.g., IL-10, TGF-β1 alongside IFN-γ, and MCP-1) suggests a dysregulated macrophage state that sustains inflammation without resolution.^14,50^ Mechanistically, the emergence of this hypersecretory phenotype may involve cholesterol-driven changes in membrane organization, nuclear receptor signaling, or mitochondrial metabolism, as suggested by prior studies.^6,50^ While these pathways were not directly tested here, our metabolomic data are consistent with mitochondrial involvement, and further work will be required to establish causality.

In summary, cholesterol conditioning disrupts canonical M2 polarization by reducing expression of key repair and regulatory markers while amplifying cytokine secretion. Similar effects were observed under M1-polarizing conditions (Figure 4), indicating that cholesterol consistently drives macrophages into a hypersecretory state with potential consequences for maladaptive immune remodeling in cholesterol-rich environments.

## 4 General Discussion and Conclusion

This study provides evidence that chronic exposure to free cholesterol reprograms macrophages toward a noncanonical, hypersecretory phenotype, functionally uncoupled from classical surface marker expression. These findings underscore the importance of considering metabolic and lipid-related cues in shaping macrophage behavior. At the monocyte stage, cholesterol exposure already imposed a measurable cellular burden. We observed a reduction in cell count above 3.06 mg/mL, an early decline in ROS signals beginning at 2.5 mg/mL, and metabolic remodeling characterized by free cholesterol accumulation, TCA intermediate depletion, redox imbalance, and compensatory glycolysis. These findings suggest that cholesterol stress compromises mitochondrial activity and redox buffering capacity, creating a metabolic landscape primed for downstream functional reprogramming. Upon differentiation into uncommitted M0 macrophages, cholesterol-loaded monocytes showed minimal phenotypic changes based on clustering analysis, though we noted a modest expansion of a regulatory antigen-presenting subset and slight increases in IL-10, IFN-γ, and IP-10. While subtle, these findings suggest cholesterol-treated macrophages for altered immune responses, even in the absence of polarization cues. Under M1-polarizing conditions, cholesterol-treated macrophages failed to fully acquire a classical M1 phenotype, as indicated by reduced CD80, CD86, and HLA-DR expression. Yet, cytokine secretion was paradoxically enhanced, with elevated levels of IL-12p70, IFN-γ, IL-17A, MCP-1, IL-10, and IL-4. Similarly, cholesterol disrupted canonical M2 polarization by reducing surface expression of CD206, CD105, and CD163 while increasing secretion of both regulatory cytokines (IL-10, TGF-β1) and inflammatory mediators (IFN-γ, IL-12p70, IL-17A). Rather than adopting a defined M1 or M2 phenotype, these cells appear to display functional dysregulation, which may impair their ability to coordinate coherent immune responses. This hybrid cytokine signature could lead to persistent low-grade inflammation, delayed inflammatory resolution, or ineffective immune surveillance, where chronic lipid exposure alters macrophage programming.

Across both M1 and M2 conditions, cholesterol conditioning consistently redirected macrophages toward a noncanonical hypersecretory phenotype. Cytokine levels from M1 and M2 conditions were replotted for direct comparison in Supplementary Figure S6. To determine whether this hypersecretory state extended beyond our observed cytokines, we quantified total protein secretion. Indeed, cholesterol-treated M1 and M2 macrophages showed a significant increase in overall secretory output, while cholesterol-exposed M0 macrophages did not (Figure S5). Although the mechanism driving this functional uncoupling was not explored in depth, it is plausible that cholesterol may promote the hypersecretory phenotype through both shared and polarization-specific signaling pathways. To begin probing this, we performed preliminary assessments of NFκB expression and the phosphorylation status of STAT3 and STAT6. However, no significant differences were observed between control and cholesterol-treated M0, M1, or M2 macrophages (Supplementary Figure S8). While activation of these pathways has been reported in response to modified lipoproteins such as AcLDL, our use of soluble, unesterified cholesterol in a 3D culture context may engage distinct signaling mechanisms,^26,52^ warranting further investigation into alternative regulatory pathways. Additionally, lipid peroxidation, ER stress markers, and transcriptional regulators may be a key consequence of excessive cholesterol and may play a central role in driving the hypersecretory macrophage phenotype observed in this study. However, this requires further investigation.

While no in vitro macrophage model perfectly replicates primary human immune cells, THP-1-derived macrophages offer a genetically uniform, phenotypically stable, and experimentally tractable system that is well-suited for mechanistic studies and high-throughput applications. Their use minimizes biological variability and allows for controlled analysis of cholesterol-induced phenotypes, making them a robust platform for investigating macrophage responses under defined lipid conditions. THP-1 cells have also been widely used in studies of cancer biology,^53^ wound healing,^9^ and biomaterials testing,^40^ consistently demonstrating promising results across diverse experimental settings. For a more physiologically relevant context, future studies should include primary human monocyte-derived macrophages from donors with characterized lipid profiles to validate the uncoupling of surface markers and cytokine secretion observed in this study. Classifying donors based on their individual cholesterol levels would enable evaluation of whether baseline lipid status influences macrophage polarization and secretory phenotypes. However, isolating and working with peripheral blood mononuclear cells (PBMCs) presents several challenges. Donor-to-donor variability, driven by factors such as diet, genetics, race/ethnicity, and metabolic background, can significantly impact lipid metabolism and immune responses. In the absence of detailed donor lipid profiles, baseline differences in cholesterol exposure and metabolic preconditioning could substantially influence macrophage phenotype and functional responses, thereby confounding interpretation of cholesterol-specific effects. To avoid this variability and to ensure a uniform and well-controlled starting condition, we elected to use THP-1 cells, which allowed systematic preconditioning in a defined high-cholesterol environment and enabled direct attribution of the observed phenotype–function uncoupling to cholesterol exposure.

## Supplementary Material



## Notes

Figure S1: Heatmap of intracellular metabolites in control and cholesterol-treated cells; Figure S2: ATP levels, measured by NMR-based metabolomic analysis; Figure S3: Effects of cholesterol exposure on mitochondrial membrane potential in THP-1 cells; Figure S4: Differentiation markers in monocytes vs M0 differentiated cells under control and cholesterol-treated conditions; Figure S5: Compares population-level surface marker expression between control and cholesterol-treated M0 macrophages; Figure S6: Direct comparison of cytokine expression between M1 and M2 macrophages under control and cholesterol-treated conditions; Figure S7: Total protein quantification profiles in culture supernatants of M0, M1, and M2 macrophages; Figure S8: Cholesterol treatment did not significantly alter NFκB expression or STAT3 and STAT6 activation across macrophage phenotypes (PDF)
