# Supplementary material for: Chronic Cholesterol Exposure Disrupts Macrophage Polarization and Cytokine Secretion in a 3D Microenvironment
Source: ACS Omega. 2026 Feb 5;11(6):9408–20. doi: 10.1021/acsomega.5c09175 (PMC12917798; doi:10.1021/acsomega.5c09175)
Supplement: Supplementary file 1 [file ao5c09175_si_001.pdf]

Supplementary Information

Chronic Cholesterol Exposure Disrupts Macrophage Polarization and Cytokine Secretion in a 3D Microenvironment

Aliyaa Ali Alzaabi[1,*], Dheyab Saleh Abubaker[1,*], Jiranuwat Sapudom[1,2], Yamanappa Hunashal[3,4], Fabio Piano[3,4,5], Jeremy Teo[1,2,6,#]

[1] Laboratory for Immuno Bioengineering Research and Applications, Division of Engineering, New York University Abu Dhabi, Abu Dhabi, 129188 UAE

[2] Department of Biomedical Engineering, Tandon School of Engineering, New York University, NY, 11201 USA

[3] Biology Program, Science Division, New York University Abu Dhabi, Abu Dhabi, 129188 UAE

[4] Center for Genomics and Systems Biology, New York University Abu Dhabi, Abu Dhabi, 129188 UAE

[5] Department of Biology, Center for Genomics and System Biology, New York University, NY, 11201 USA

[6] Department of Mechanical Engineering, Tandon School of Engineering, New York University, NY, 11201 USA

* Contributed equally to this work

# Corresponding Author (jeremy.teo@nyu.edu)

Supplementary Table 1: Antibodies used in this study

Marker	Color/Format	Host/Target	Isotype	Clone	Company catalog No.
CD105	Alexa Fluor 647	Mouse anti-Human	IgG1 κ	43A3	Biolegend, 323212
CD206	Brilliant Violet 510	Mouse anti-Human	IgG1 κ	15-2	Biolegend, 321138
HLA-DR	Brilliant Violet 421	Mouse anti-Human	IgG2a, κ	L243	Biolegend, 307636
CD11b	Alexa Fluor 700	Mouse anti-Human	IgG1 κ	ICRF44	Biolegend, 301356
CD163	APC/Fire 750	Mouse anti-Human	IgG1 κ	GHI/61	Biolegend, 333634
HLA-DR	Alexa Fluor 488	Mouse anti-Human	IgG2a, κ	L243	Biolegend, 307656
CD80	Brilliant Violet 711	Mouse anti-Human	IgG1 κ	2D10	Biolegend, 305236
CD86	Brilliant Violet 605	Mouse anti-Human	IgG1 κ	BU63	Biolegend, 374214
CD83	PE/Cyanine 5	Mouse anti-Human	IgG1 κ	HB15e	Biolegend, 305310
TLR4	Brilliant Violet 421	Mouse anti-Human	IgG2a, κ	HTA125	Biolegend, 312811
CD11b	PE/Dazzle 594	Mouse anti-Human	IgG1 κ	ICRF44	Biolegend, 301347
CD163	PE/Cy7	Mouse anti-Human	IgG1 κ	GHI/61	Biolegend, 333614
CD11b	Brilliant Violet 650	Mouse anti-Human	IgG1 κ	ICRF44	Biolegend, 301336
CD14	APC/Cy 7	Mouse anti-Human	IgG1 κ	63D3	Biolegend, 367108
CD68	PE-Cy7	Mouse anti-Human	IgG1 κ	eBioY1/82A	Invitrogen, 25-0689-42
NFkB p50	-	Mouse anti-Human	IgG1 κ	4D1	Biolegend, 616701
Goat anti-Mouse IgG (H&L)	DyLight 594	Goat anti-Mouse	IgG1	-	Immunereagents, GtxMu-003-D594NHSX
STAT3	Alexa Flour 488 phospho (Tyr705)	Mouse anti-Human	IgG1 κ	13A3-1	Biolegend, 651005
STAT6	Alexa Flour 647 phospho (Tyr641)	Mouse anti-Human	IgG1 κ	A15137E	Biolegend, 686011

Supplementary Table 2: Differential metabolite levels in control versus cholesterol-treated cells (Fold Change > 1.5, $p < 0.05$).

Metabolites increased in control (decreased with cholesterol treatment)	Metabolites increased in cholesterol-treated cells
Isocitrate	3-Methyl-2-oxovalerate
Glycylproline	Isopropanol
Aspartate	Citraconate
trans-4-Hydroxy-L-proline	Maleate
Methylsuccinate	Guanidinosuccinate
Glutaric acid monomethyl ester	
Succinate	
O-Phosphocholine	
Homoserine	
Choline	
Hydroxyacetone	
Formate	
ATP	
sn-Glycero-3-phosphocholine	
Guanidoacetate	
Myo-Inositol	
Glucarate	
Xanthosine	
Phenylalanine	

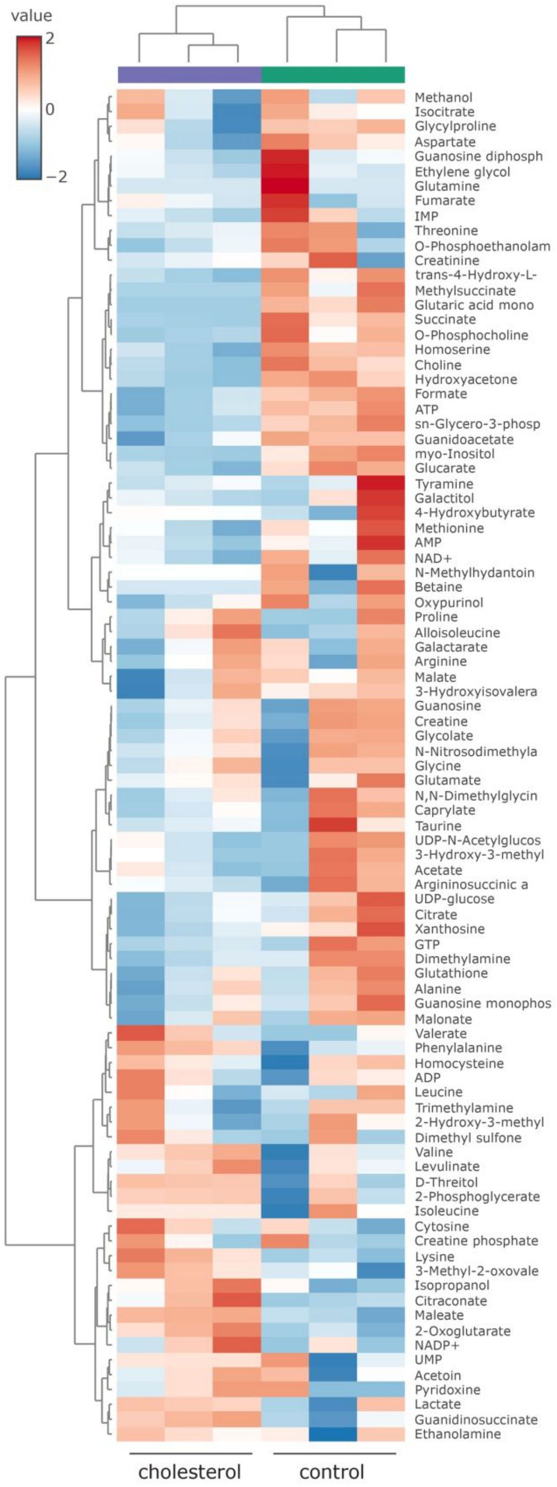

Supplementary Figure S1: Heatmap of intracellular metabolites in control and cholesterol-treated cells. Red indicates relative upregulation and blue indicates downregulation. Data was replotted from Figure 2C for better visualization. Statistical analysis was performed using Student's *t*-test; $p < 0.05$ (*). Data represent triplicate.

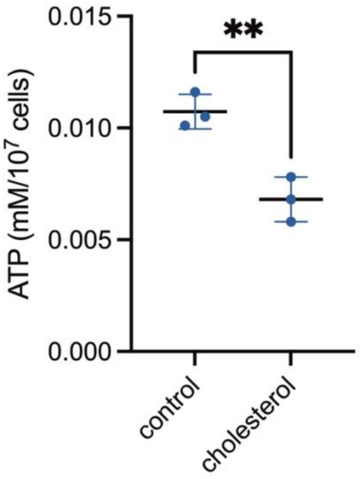

Supplementary Figure S2: ATP levels measured by NMR-based metabolomic analysis. ATP abundance was assessed using NMR-based metabolomic profiling from THP-1 with and without cholesterol exposure after 3 passages of culture. Data are presented as ATP amount normalized to internal standards. Quantitative results represent the mean ± SD from three independent experiments (n = 3). Statistical significance was determined using Student's *t*-test; $*p < 0.05$.

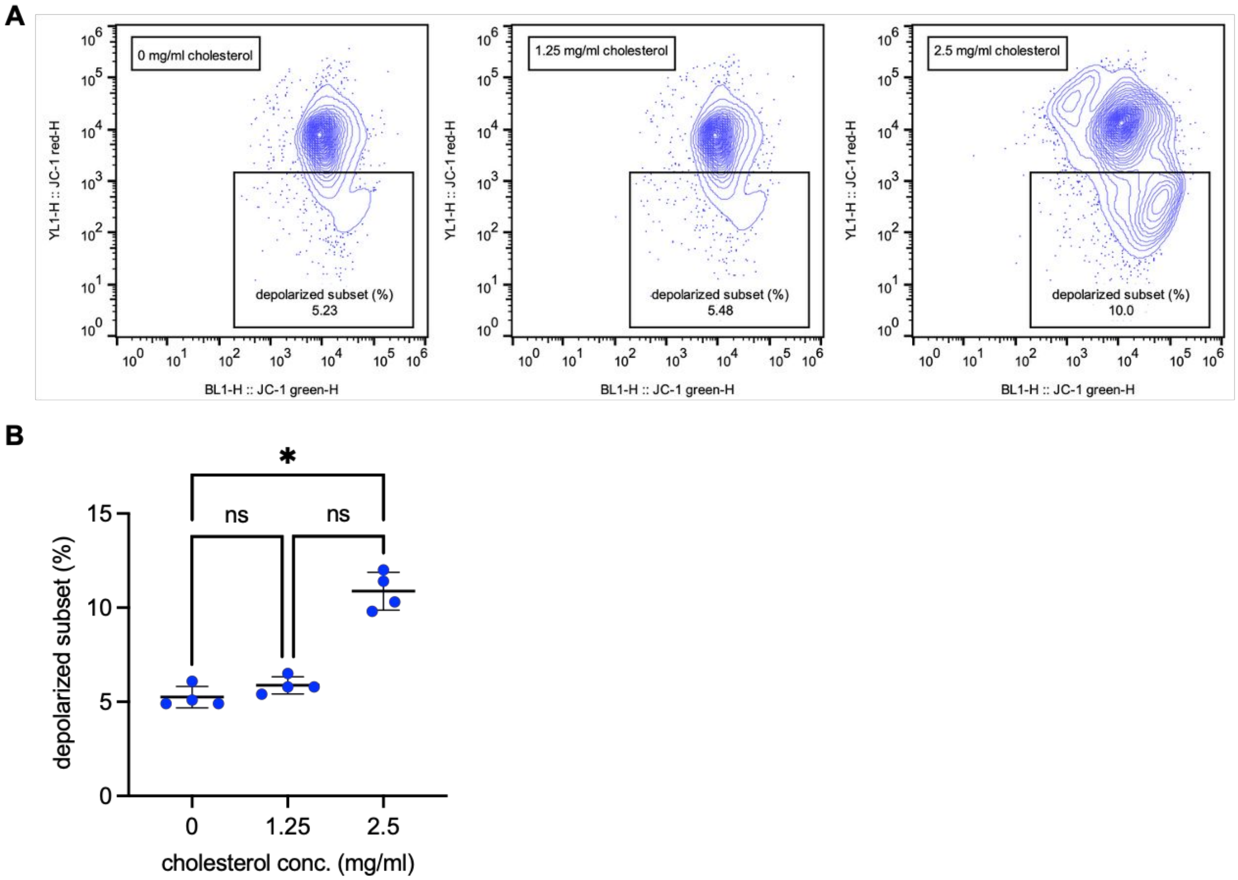

Supplementary Figure S3: Effects of cholesterol exposure on mitochondrial membrane potential in THP-1 cells. (A) Representative flow cytometry plots of JC-1 staining in THP-1 cells after three passages of culture with 0, 1.25, or 2.5 mg/mL cholesterol. JC-1 green (monomer) and red (aggregate) fluorescence indicate depolarized and polarized mitochondria, respectively. The boxed gate denotes the depolarized subset, expressed as a percentage of total cells. (B) Quantitative analysis of the percentage of JC-1–positive depolarized cells from four independent experiments. Data are presented as mean ± SD. Statistical significance was determined using Kruskal-Wallis test;*$p < 0.05$.

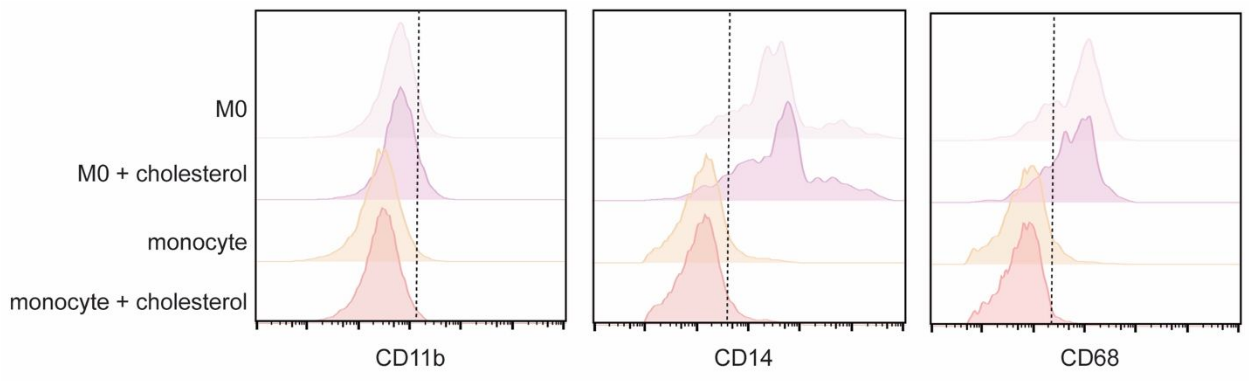

Supplementary Figure S4: Differentiation markers in monocytes versus M0 differentiated cells in control and cholesterol-treated cells. Representative histogram plot shows the differentiation markers CD11b, CD14, and CD68 in THP-1 cells and in differentiated M0 cells.

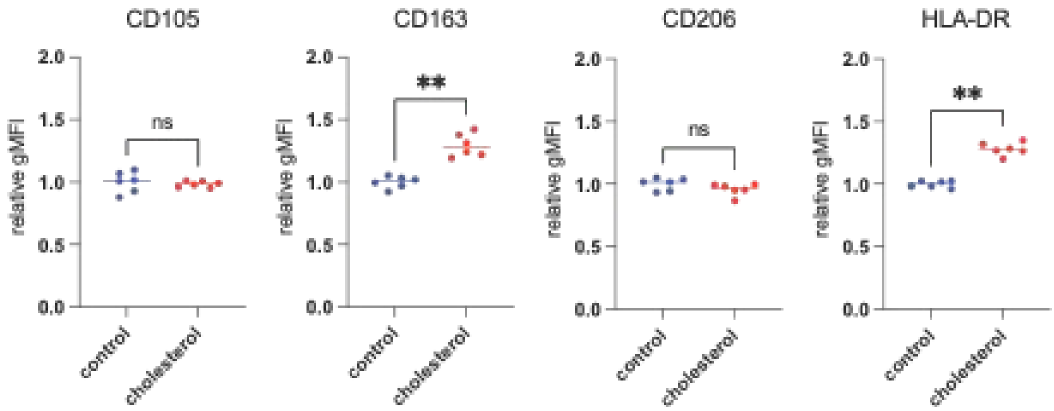

Supplementary Figure S5: Population-level surface marker expression comparing control and cholesterol-treated M0 macrophages. Values were normalized to control. Data was replotted from Figure 3E. Data are presented as mean ± SD. Statistical analysis was performed using Student's *t*-test; $p < 0.01$ (**). Experiments were performed in 6 replicates.

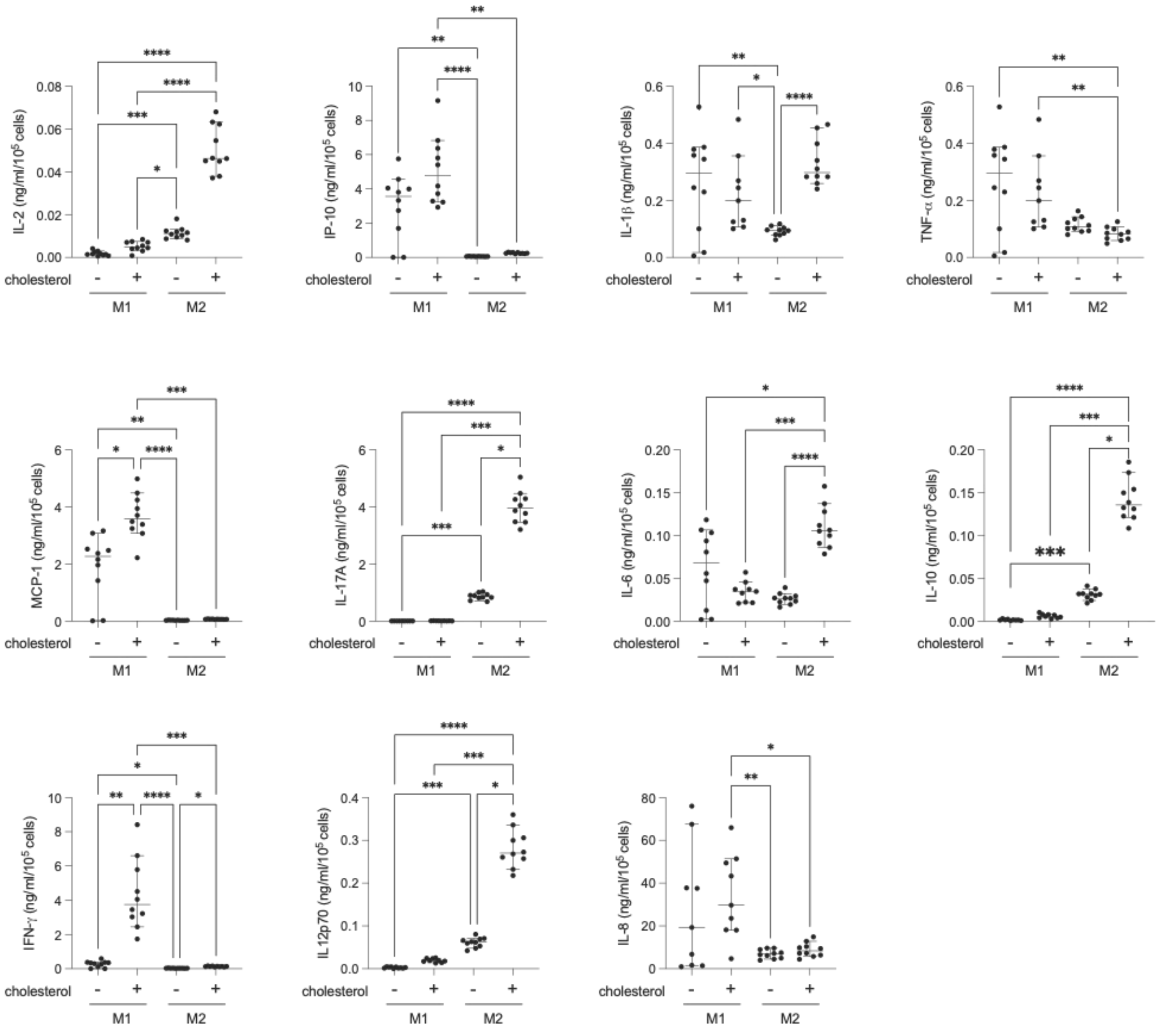

Supplementary Figure S6: Direct comparison of cytokine expression between M1 and M2 control and cholesterol-treated macrophages. Cytokine secretion profiles were measured in culture supernatants of M1 and M2 macrophages using a bead-based multiplex immunoassay. Data were replotted from Figures 4F and 5F to enable direct comparison. Statistical differences between groups were assessed using the Kruskal–Wallis test, followed by Dunn's post hoc test. Significance levels are indicated as follows: $p < 0.05$ (*), $p < 0.01$ (**), $p < 0.001$ (***), and $p < 0.0001$ (****). Experiments were performed in at least 6 replicates.

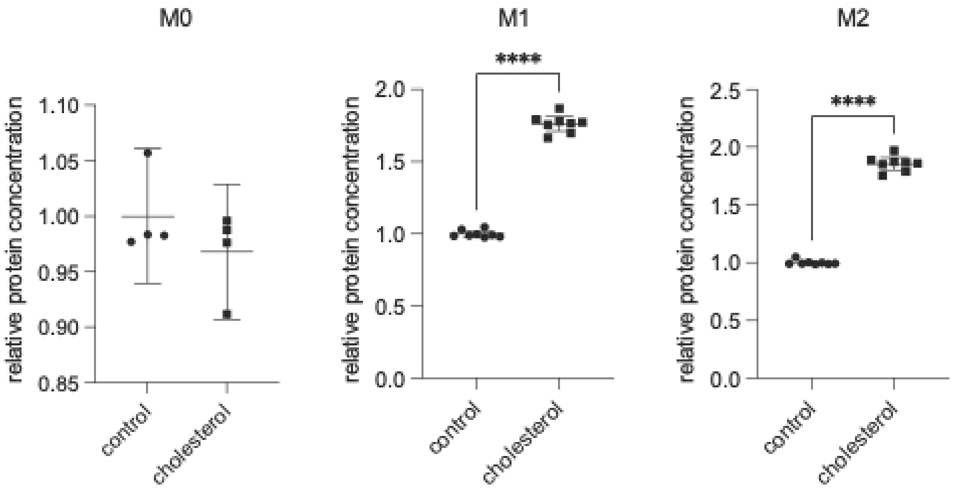

Supplementary Figure S7: Total protein quantification profiles in culture supernatants of M0, M1, and M2 macrophages. Protein Quantification using Nanodrop direct absorbance measurements at 280 nm. Values were normalized to control. Data are presented as mean ± SD. Statistical differences between groups were assessed using Student's t-test. Significance levels are indicated as: $p < 0.0001$ (****). Experiments were performed in at least 4 replicates.

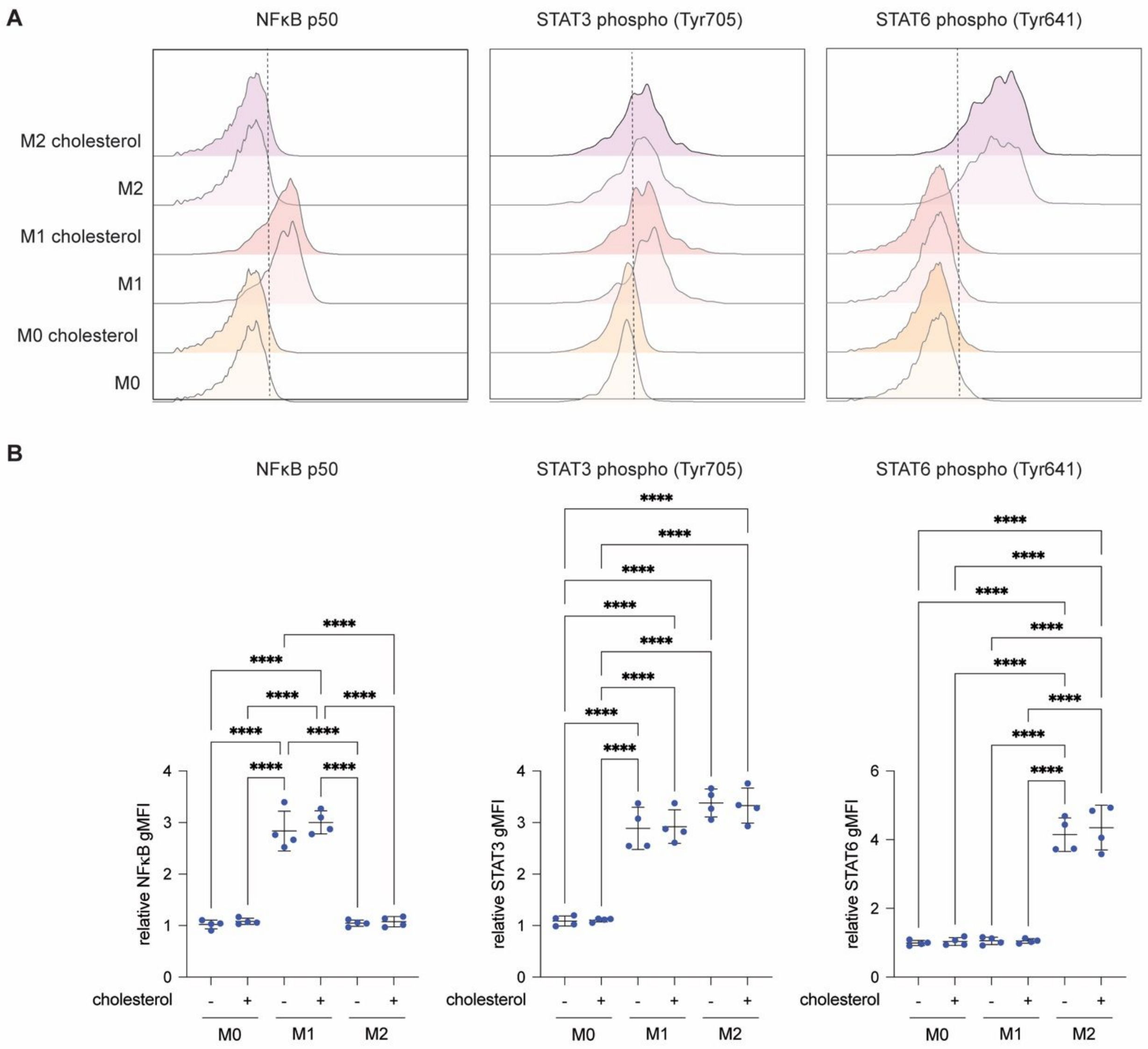

Supplementary Figure S8: Cholesterol treatment did not significantly alter the expression level of NFκB, or the activation of STAT3 and STAT6 across macrophage phenotypes. (A) Representative flow cytometry data showing NFκB p50, STAT3 phosphorylation at Tyr705 (STAT3 (Tyr705)), and STAT6 phospholylation at Tyr641 (STAT6 (Tyr641)). **(B)** Quantitative analysis of normalized geometric mean of fluorescence intensity (gMFI) of NFκB**,** STAT3 (Tyr705) and STAT6 (Tyr641). Consistent with their known polarization profiles, NFκB p50 levels were highest in M1 macrophages, STAT6 (Tyr641) phosphorylation was most pronounced in M2 macrophages, and STAT3 (Tyr705) phosphorylation was comparably elevated in both M1 and M2 macrophages relative to M0. Experiments were performed in 4 replicates.
